# Familiarity With and Use of Clinical Prediction Models in Nephrology Practice in Poland

**DOI:** 10.1016/j.ekir.2026.107073

**Published:** 2026-09-08

**Authors:** Jakub Ruszkowski, Alicja M. Dębska-Ślizień, Jolanta Małyszko

**Affiliations:** 1 Department of Nephrology, Transplantology and Internal Medicine, Faculty of Medicine, Medical University of Gdańsk, Poland; 2 Department of Nephrology, Dialysis and Internal Medicine, Warsaw Medical University, Warsaw, Poland

**Keywords:** implementation, predictive learning models, prognosis, renal insufficiency, risk assessment

## Introduction

Chronic kidney disease (CKD) affects approximately 14% of adults worldwide, yet its course is heterogeneous, with markedly different rates of progression to kidney failure.^1^^,^S1 Clinical prediction models address this heterogeneity by estimating individualized risk to guide nephrology referral, selection of disease-modifying therapy, timing of vascular access creation, and shared decision-making, particularly in elderly patients in whom competing mortality risk may exceed kidney failure risk.^2^

Recognizing this utility, the 2024 Kidney Disease: Improving Global Outcomes (KDIGO) guideline for CKD recommends validated prediction models in stages G3–G5 to estimate kidney failure risk, foremost the Kidney Failure Risk Equation (KFRE), a grade 1A recommendation.^3^^,^^4^ It also endorses disease-specific, externally validated prediction tools: the International IgA nephropathy Prediction Tool for IgA nephropathy, and the Mayo Imaging Classification and PROPKD score for autosomal dominant polycystic kidney disease, detailed in the respective KDIGO guidelines.^3^^,^S2^,^S3 Despite strong evidence, adoption in routine nephrology practice appears suboptimal.^2^^,^^5^

We therefore assessed familiarity with and use of clinical prediction models among nephrologists practicing in Poland, identified implementation barriers, and explored training preferences. We conducted a cross-sectional, anonymous, 17-item survey of board-certified nephrologists and trainees practicing in Poland (August–October 2025), covering knowledge and use of prediction models, clinical needs, barriers, attitudes, competencies, and training preferences. Full methods are provided in the Supplementary Methods.

## Results

A total of 131 nephrologists responded, representing at most about 15% of actively practicing Polish nephrologists (Supplementary Methods).S7–S10 Most were women (63.4%) and worked primarily in university/academic hospitals (61.8%), mainly managing non-dialysis CKD G3–G5 (84.7%) and hemodialysis (75.6%) patients (Supplementary Tables S1 and S17). Although 67.9% (95% CI: 59.5–75.3) reported at least good familiarity with the 2024 KDIGO CKD guideline, only 12.1% (95% CI: 3.2–21.5) used KFRE regularly, 40.3% used it rarely, and 47.6% did not use it or were unaware of it. Familiarity with the guideline correlated strongly with KFRE use (Supplementary Figure S1 and Supplementary Table S14; γ = 0.70; τ_b_ = 0.48; both *P* < 0.001).

The most frequently used tools were not nephrology-specific but were learned during undergraduate training: CHA_2_DS_2_-VASc/CHA_2_DS_2_-VA (84.6%) and HAS-BLED (77.2%) (Figure 1). Nephrology-specific tools were used far less often: KFRE (39.0%), the International IgA nephropathy Prediction Tool (32.5%), the Mayo Imaging Classification for autosomal dominant polycystic kidney disease (26.0%), and the Renal Insufficiency in Nephrology REIN score (15.4%); only 62.6% (95% CI: 53.8–70.6) used at least 1 nephrology-specific tool, and 5.7% used none. Use of the disease-specific tools was concentrated in academic centers, whereas KFRE use did not differ by setting (Supplementary Tables S2–S3). In multivariable analysis, guideline familiarity was the only independent predictor of KFRE use; academic setting independently predicted use of the disease-specific tools but not KFRE (Supplementary Tables S4–S7).

**Figure 1:**
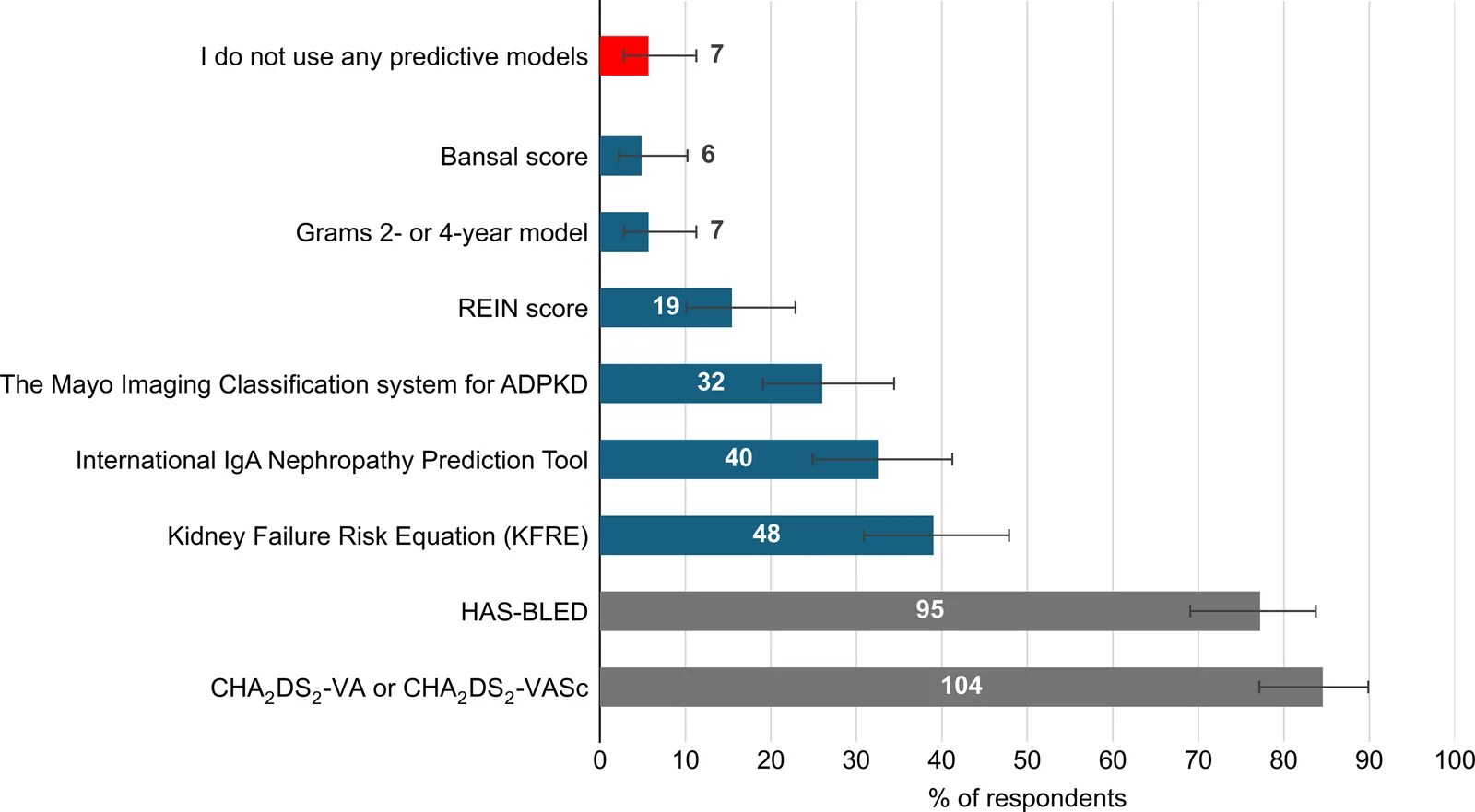
Use of clinical prediction models in practice among respondents (*n* = 123). Bar length represents the percentage of respondents (error bars are 95% Wilson confidence intervals); numbers within bars indicate the absolute number of respondents.

A striking discrepancy emerged between perceived needs and actual use. Respondents most often selected progression to kidney failure (72.5%), catheter-related bloodstream infection (64.1%), and arteriovenous fistula dysfunction (56.5%) as the outcomes most useful to predict (Table 1). Yet among those identifying kidney failure progression as useful, only 41.6% used KFRE and 5.6% used Grams models. Priorities diverged by setting (Table 1; Supplementary Results).

**Table 1:** Outcomes selected by respondents (*n* = 131) as useful to predict, overall and by practice setting

Outcome selected as useful to predict	All	Academic	Nonacademic	*P*^a^
Progression to kidney failure requiring kidney replacement therapy	95 (72.5)	53 (65.4)	42 (84.0)	0.02
Catheter-related bloodstream infection in hemodialysis patients	84 (64.1)	43 (53.1)	41 (82.0)	< 0.001
Arteriovenous fistula dysfunction	74 (56.5)	33 (40.7)	41 (82.0)	< 0.001
Cardiovascular events in dialysis patients	68 (51.9)	38 (46.9)	30 (60.0)	0.15
Severe infections after immunosuppressive treatment in glomerulonephritis	65 (49.6)	51 (63.0)	14 (28.0)	< 0.001
Severe infections after kidney transplantation	60 (45.8)	45 (55.6)	15 (30.0)	0.004
Kidney transplant rejection	59 (45.0)	48 (59.3)	11 (22.0)	< 0.001
Bleeding after kidney biopsy	56 (42.7)	43 (53.1)	13 (26.0)	0.002
Cardiovascular events in nondialysis CKD patients	49 (37.4)	30 (37.0)	19 (38.0)	0.92
Cardiovascular death in dialysis CKD patients	48 (36.6)	25 (30.9)	23 (46.0)	0.08
Peritoneal dialysis-associated peritonitis in peritoneal dialysis patients	48 (36.6)	34 (42.0)	14 (28.0)	0.11
All-cause mortality in dialysis CKD patients	46 (35.1)	27 (33.3)	19 (38.0)	0.59
Death in kidney transplant patients	42 (32.1)	32 (39.5)	10 (20.0)	0.02
Hospitalization in patients with CKD on dialysis	39 (29.8)	20 (24.7)	19 (38.0)	0.11
Cardiovascular death in patients with non-dialysis CKD	37 (28.2)	25 (30.9)	12 (24.0)	0.40
All-cause mortality in patients with non-dialysis CKD	32 (24.4)	24 (29.6)	8 (16.0)	0.08
Hospitalization in patients with non-dialysis CKD	28 (21.4)	19 (23.5)	9 (18.0)	0.46

Values are *n* (%).

CKD, chronic kidney disease.

a *P*-value based on Pearson’s chi-square test of independence (unadjusted for multiple comparisons).

The dominant barriers were organizational and technological rather than financial: lack of time during visits (77.1%), lack of institutional support (63.4%), absence of electronic health record (EHR) integration (74.0%), and the lack of a single website giving access to multiple tools (58.0%); by contrast, lack of additional payment was a barrier for only 14.5% of respondents (Supplementary Tables S8–S9). Attitudes were positive: most respondents trusted model results (84.0%), agreed models increase decision confidence (90.1%), and agreed they improve care quality (87.8%) (Supplementary Table S10), with no significant differences by gender, experience, or setting. On the 5-point interpretive-competence scale, only 15.3% chose the highest option — being able to interpret results and explain them to patients (Supplementary Table S11). Non-users (11.5%) more often worked outside academic centers (66.7% vs. 33.9%, *P* = 0.04) and rated their familiarity with the guideline as poor (Supplementary Tables S11–S13).

The most requested training topics were practical use (52.7%), interpreting results (45.0%), and integration into decision-making (42.7%); self-paced online courses were the preferred format (64.1%), though reaching most nephrologists would require combining several formats (Supplementary Results and Supplementary Figure S2). Notably, every non-user expressed interest in training, suggesting the gap is addressable.

## Discussion

This survey of 131 respondents reveals a gap between guideline recommendations and practice. Regular KFRE use was low (12.1%) despite positive attitudes, a grade 1A KDIGO recommendation, and extensive European validation.^3^^,^S4^,^S5 This gap mirrors slow uptake elsewhere: at Johns Hopkins, KFRE documentation reached only about 25% of outpatient nephrology notes a year after Epic integration, indicating that EHR integration alone is insufficient.^5^

Attitudes and perceived barriers did not explain who used KFRE: among respondents who knew the guideline well and endorsed prediction models, users and non-users reported similar barriers, and only knowledge and self-rated interpretive skill tracked use (Supplementary Tables S14–S16).

The strong link between guideline familiarity and use suggests education can help, yet access barriers persist: no Polish nephrology recommendation introduces prediction models into specialist practice, and the KFRE is named only in a primary-care documentS6 that stops at naming the tool without defining how to act on the estimate (see the Supplementary Discussion and Supplementary References,S11–S18). Practical constraints also apply: KFRE requires albuminuria testing often unavailable in Polish primary care, the IgA nephropathy tool requires biopsy histology rarely repeated, and the PROPKD score requires genetic testing seldom performed in Poland. The dominance of cardiology-derived scores reflects their earlier curricular introduction and argues for embedding nephrology-specific tools in undergraduate and postgraduate training.

Time pressure and the strong demand for EHR integration—mirroring international experience—indicate that automating risk calculation could lower barriers,^6^ because knowledge and interpretive skill appear to be more important. The mismatch between clinical needs and use is most striking for kidney failure prediction, where registry analyses show KFRE-based strategies outperforming traditional criteria for specialist referral and vascular-access planning.^7^^,^^8^ Implementation must nonetheless respect each tool's validated scope: the European Renal Best Practice commentary advocates local validation and recalibration.^9^ The KFRE has been validated in European cohorts but, to our knowledge, not in Polish cohorts; dedicated validation is therefore needed, and until then, models validated in comparable European populations remain a reasonable interim choice.

This study has several limitations. Our respondents are not a probability sample of the Polish nephrology workforce: voluntary, snowball-based recruitment precluded a response-rate calculation and favored clinicians who were more interested in prediction models, worked more often in academic, and were younger; these biases and social desirability biases make the figures upper bounds on prediction-model use in Poland. The questionnaire lacked items about consequences — whether an estimated risk ever changed a management or discharge decision (Supplementary Discussion).

In conclusion, in this sample of nephrologists practicing in Poland, clinical prediction models were used less often than their favorable reception would suggest, and what separated users from non-users was knowledge and interpretive skill rather than the near-universal organizational and technological barriers. Training was welcomed, with self-paced online courses preferred. Some of this picture is likely specific to Poland, where no specialty-level recommendation introduces prediction models into nephrology practice; the dissociation between favorable attitudes and use, the primacy of time and EHR integration over financial barriers, and training preferences are likely to generalize. Closing the gap will require education at every career stage alongside EHR integration and freely accessible online tools (Supplementary Discussion).

## Disclosure

All the authors declared no competing interests.

## Supplementary Material

Supplementary File (PDF) **Supplementary Methods. Supplementary Results. Supplementary Discussion. Supplementary References. Figure S1.** Correlation between familiarity with the 2024 KDIGO CKD guideline and frequency of KFRE use. **Figure S2.** UpSet plot of preferred training methods. **Table S1.** Study population characteristics. **Table S2.** Setting-stratified estimates. **Table S3.** Directly standardized estimates. **Table S4.** Multivariable logistic regression for KFRE use: primary model. **Table S5.** Sensitivity model for KFRE use omitting guideline familiarity. **Table S6.** Sensitivity analyses for the association between guideline familiarity and KFRE use. **Table S7.** Predictors of the use of other prediction tools. **Table S8.** Organizational barriers to implementing predictive models. **Table S9.** Technological barriers to implementing predictive models. **Table S10.** Attitudes toward prediction models. **Table S11.** Self-rated knowledge in interpreting prediction model results, by user status. **Table S12.** Characteristics comparison of non-users and users of clinical prediction models. **Table S13.** Comparison of attitudes toward prediction models between non-users and users. **Table S14.** Association of guideline familiarity, interpretive competence, attitudes and barriers with frequency of KFRE use. **Table S15.** KFRE use across the self-rated interpretive-competence scale. **Table S16.** KFRE users versus non-users within the informed and favorably disposed subgroup. **Table S17.** Reconstruction of respondent age from years of nephrology experience.

## Footnotes

Supplementary File (PDF)

**Supplementary Methods.**

**Supplementary Results.**

**Supplementary Discussion.**
**Supplementary References**. **Figure S1.** Correlation between familiarity with the 2024 KDIGO CKD guideline and frequency of KFRE use. **Figure S2.** UpSet plot of preferred training methods. **Table S1.** Study population characteristics. **Table S2.** Setting-stratified estimates. **Table S3.** Directly standardized estimates. **Table S4.** Multivariable logistic regression for KFRE use: primary model. **Table S5.** Sensitivity model for KFRE use omitting guideline familiarity. **Table S6.** Sensitivity analyses for the association between guideline familiarity and KFRE use. **Table S7.** Predictors of the use of other prediction tools. **Table S8.** Organizational barriers to implementing predictive models. **Table S9.** Technological barriers to implementing predictive models. **Table S10.** Attitudes toward prediction models. **Table S11.** Self-rated knowledge in interpreting prediction model results, by user status. **Table S12.** Characteristics comparison of non-users and users of clinical prediction models. **Table S13.** Comparison of attitudes toward prediction models between non-users and users. **Table S14.** Association of guideline familiarity, interpretive competence, attitudes and barriers with frequency of KFRE use. **Table S15.** KFRE use across the self-rated interpretive-competence scale. **Table S16.** KFRE users versus non-users within the informed and favorably disposed subgroup. **Table S17.** Reconstruction of respondent age from years of nephrology experience.
