# Supplementary material for: Familiarity With and Use of Clinical Prediction Models in Nephrology Practice in Poland
Source: Kidney Int Rep. 2026 Sep 8;11(11):107073. doi: 10.1016/j.ekir.2026.107073 (PMC13653939; doi:10.1016/j.ekir.2026.107073)
Supplement: Supplementary file 1 [file mmc1.pdf]

Familiarity with and use of clinical prediction models in nephrology practice in Poland

<u>Supplementary Material</u>

Supplementary Methods ... 2

Study Design and Setting ... 2

Participants ... 2

Survey Instrument ... 2

Recruitment ... 3

Representativeness ... 3

Statistical Analysis ... 4

Supplementary Results ... 7

Respondent Characteristics ... 7

Clinical Needs ... 7

Implementation Barriers ... 8

Attitudes Toward Prediction Models ... 8

Attitudes, Knowledge and Skill versus Barriers ... 8

Characteristics of Non-Users of Clinical Prediction Models ... 9

Training Needs and Preferences ... 9

Workforce Age Structure and Reconstructed Respondent Age ... 9

Supplementary Discussion ... 11

Clinical Recommendations on Prediction Models in Poland ... 11

Actionability of a Risk Estimate in the Polish Setting ... 11

Future Directions ... 12

Supplementary Tables ... 13

Table S1. Study population characteristics
Table S2. Key estimates stratified by practice setting
Table S3. Key estimates directly standardized to national distributions of practice setting
Table S4. Predictors of KFRE use: primary multivariable logistic regression model
Table S5. Predictors of KFRE use: sensitivity model omitting guideline familiarity
Table S6. Sensitivity analyses for the association between guideline familiarity and KFRE use
Table S7. Predictors of the use of other prediction tools
Table S8. Organizational barriers to implementing predictive models
Table S9. Technological barriers to implementing predictive models
Table S10. Attitudes toward prediction models
Table S11. Self-rated knowledge in interpreting prediction model results, by user status
Table S12. Characteristics comparison of non-users and users of clinical prediction models
Table S13. Comparison of attitudes toward prediction models between non-users and users
Table S14. Association of guideline familiarity, interpretive competence, attitudes and barriers with frequency of KFRE use
Table S15. KFRE use across the self-rated interpretive-competence scale
Table S16. Users versus non-users of the KFRE among informed and favorably disposed respondents
Table S17. Reconstruction of respondent age from years of nephrology experience

Supplementary Figures ... 21

Figure S1. Correlation between familiarity with the KDIGO 2024 CKD guideline and frequency of KFRE use
Figure S2. UpSet plot of preferred training methods

Supplementary References ... 23

Supplementary Methods

Study Design and Setting

We conducted a cross-sectional survey study among nephrologists practicing in Poland between August and 20 October 2025. Since this was a voluntary and anonymous survey among nephrologists with no patient involvement, approval from an ethics committee was not required. This was confirmed by the Bioethics Committee for Scientific Research at the Medical University of Gdańsk (confirmation received August 8, 2025).

Participants

Eligible participants included board-certified nephrologists and physicians in nephrology training programs working in any clinical setting in Poland, including university/academic hospitals, non-university hospitals, dialysis centers, and outpatient clinics. No exclusion criteria were applied. A convenience sampling approach was used; no formal sample size calculation was performed a priori.

Survey Instrument

A 17-item questionnaire was developed by the research team covering six thematic domains: knowledge and current use of KDIGO guidelines and prediction models (Questions 1–4); clinical needs for risk prediction (Question 5); implementation barriers (Questions 6–7); attitudes and trust toward prediction models (Question 8); competencies and training needs (Questions 9–11); and respondent characteristics (Questions 12–17). The questionnaire was developed in Polish and administered via Google Forms. Questions used various response formats, including Likert scales and multiple-choice, and demographic items were placed last

Items were generated from a structured review of the recommendations whose uptake was to be measured — the KDIGO 2024 CKD guideline and the disease-specific KDIGO guidelines for IgA nephropathy and ADPKD[3,S2,S3] — and of the implementation-science literature on barriers to adopting clinical decision support. Barrier and attitude items were restricted to determinants documented in that literature and modifiable by a professional society, a payer or a hospital — guideline familiarity, interpretive skill, electronic health record integration, availability of a single access platform, consultation time, institutional procedures and reimbursement — with instrument length held to a target completion time under ten minutes. Wording and response options were adapted to the organization and financing of Polish nephrology services. Two non-nephrology prediction tools taught in undergraduate education and used routinely in internal medicine (CHA_2DS_2-VASc/CHA_2DS_2-VA and HAS-BLED) were

included in the tool checklist as an internal benchmark. The draft was revised iteratively and then reviewed independently by the two senior co-authors, both academic nephrologists holding national roles in the specialty, for relevance, clarity, completeness and absence of leading wording, and revised accordingly. The instrument was not piloted outside the author group. It was designed as a set of stand-alone descriptive items rather than as a psychometric scale; no latent constructs were assumed, and consequently no factor analysis, internal-consistency coefficient or test–retest reliability assessment was undertaken. Response validity was instead addressed by prespecified internal-consistency checks between the three items measuring model use (Q2–Q4), described below. Self-reported use was not verified against records of actual practice.

Recruitment

Participants were recruited using a snowball sampling strategy via multiple channels, including the Young Nephrologists Club of Poland, provincial nephrology consultants, dialysis unit directors, and professional networks. Initial invitations were distributed via email and WhatsApp, with recipients encouraged to forward the survey link to other eligible nephrologists. Due to this recruitment approach, the total number of nephrologists who received the invitation cannot be determined, and the response rate cannot be calculated. Participation was voluntary and anonymous.

Representativeness

Because recruitment was by snowball sampling, no response rate could be computed and the sample is not a probability sample of the workforce. To place the 131 respondents against a national denominator, three official or semi-official sources were used. The Central Register of Physicians of the Republic of Poland (Centralny Rejestr Lekarzy RP), maintained by the Supreme Medical Council (Naczelna Rada Lekarska), recorded 1,479 physicians holding the adult nephrology specialty on 31 December 2024, of whom 1,458 were registered as practising medicine.[S7] The Supreme Audit Office (Najwyższa Izba Kontroli), drawing on Ministry of Health data, reported 1,457 professionally active nephrologists as of 30 September 2024.[S8] The number who actively practise the specialty is smaller and not officially recorded: the National Consultant in Nephrology has estimated it at only about 800–950 (reported as 800–900 to a parliamentary subcommittee in March 2025 and as 900–950 in the Supreme Audit Office's 2025 report).[S8,S9] The register counts everyone who has ever certified in nephrology, including physicians who have retired from the specialty or who work mainly in other fields, so the 131 respondents correspond to approximately 9% of all specialty holders (131/1,479) and to 13.8–

16.4% (131 of 950 to 131 of 800) of the estimated 800–950 in active practice. Both figures are upper estimates of coverage, because physicians in nephrology training were eligible but are not separately enumerated nationally; in our sample, 28.2% reported fewer than five years of experience or being in training.

The national distribution of practice settings is not published. Counts reported by the Ministry of Health to parliament in September 2023 record 1,069 nephrologists working in dialysis units against 764 in inpatient nephrology (including pediatric nephrology); these are physician–place-of-service records rather than distinct individuals and cannot be summed, but they establish that dialysis is at least as large a workplace for Polish nephrologists as hospital nephrology departments, whereas only 18.3% of our respondents worked mainly in a dialysis setting.[S10] For the academic share specifically, we reconstructed the number of units delivering nephrology care with an academic affiliation — approximately 20 university departments of nephrology and a further 7–10 clinical wards of regional hospitals serving as teaching bases, some 27–30 units in all — employing perhaps 200–300 nephrologists, or roughly 21–38% of the 800–950 estimated to practice, against 61.8% of our respondents. This reconstruction is our own approximation from publicly available institutional data, not a published statistic, and no analysis rests on any single value within it; key estimates were instead standardized across a range of academic shares (Table S3).

Statistical Analysis

Descriptive statistics were used to summarize respondent characteristics and survey responses. Categorical variables were presented as frequencies and percentages. For survey outcome variables, 95% confidence intervals (95% CIs) were calculated: the Wilson method was used for single proportions and the Sison–Glaz method for multinomial proportions. Calculations were performed with the BinomCI and MultinomCI functions from the DescTools package (version 0.99.60) in R. Associations between ordinal variables were quantified with Goodman and Kruskal's gamma (γ) and Kendall's tau-b (τ_b) also from DescTools; their confidence intervals were obtained from the asymptotic (Goodman–Kruskal) standard error. Comparisons between groups were performed using chi-square tests or Fisher's exact test, as appropriate, in jamovi. Where multiple group comparisons were made within an analysis, P-values were additionally adjusted for the false discovery rate using the Benjamini–Hochberg method; both unadjusted and adjusted P-values are reported. Respondents were classified as "non-users of clinical prediction models" if they selected "I do not use any predictive models" in Question 3 or indicated "I don't use them at all" in Question 4.

Data quality was assessed using internal consistency checks between items measuring use of predictive models (Q2–Q4). Responses were flagged as inconsistent under three conditions: (a) a respondent reported regular use of KFRE in the frequency question (Q2) but did not select KFRE in the multiple-choice checklist (Q3) [n = 4]; (b) a respondent reported not knowing or not using KFRE in the frequency question (Q2) but selected KFRE in the checklist (Q3) [n = 3]; and (c) a respondent selected specific predictive models AND the mutually exclusive option "I do not use any predictive models" within the same checklist question (Q3) [n = 2]. No inconsistent responses were identified between Q2 and Q4.

In total, 8 unique respondents with inconsistent responses were identified (6.1% of the sample). For inconsistencies (a) and (b), responses to Q2 and Q3 were excluded from relevant analyses while retaining these respondents' data for all other survey items (item-level exclusion). For inconsistency (c), one respondent's selection of KFRE was removed from Q3 as it contradicted their consistent pattern of non-use across Q2 and Q4, while retaining their "I do not use any models" response. The other respondent exhibited irreconcilable contradictions between selecting specific non-nephrological prediction models (HAS-BLED, CHA_2DS_2-VASc/CHA_2DS_2-VA) in Q3 and reporting complete non-use in Q4; their responses to Q3 and Q4 were excluded from relevant analyses while retaining their data for all other survey items.

Cumulative coverage of training formats was assessed among respondents interested in training (those who did not decline training); beginning with the most frequently selected format, formats were added one at a time in the order that maximized the number of additional respondents reached (a greedy approach), and overlap among formats was visualized with an UpSet plot.

Multivariable analysis. Predictors of KFRE use were examined by multivariable logistic regression. The outcome was any reported KFRE use (Q2: regular or rare use) versus no use, among respondents with internally consistent answers to Q2–Q4 and complete covariate data (n = 123, 65 events). Six covariates were prespecified and entered simultaneously — practice setting, sex, years of nephrology experience, weekly CKD patient volume, access to a fully integrated electronic health record, and at least good familiarity with the KDIGO 2024 guideline — with no stepwise selection. Models were fitted by maximum likelihood, and odds ratios are reported with profile-likelihood confidence intervals, except where complete separation or small event counts required Firth penalized likelihood (logistf package), for which penalized profile-likelihood intervals are given. Sensitivity analyses re-estimated the same association under a different estimator or a different operationalization of the same outcome: Firth

penalized likelihood applied to the primary model, a non-parametric bootstrap (1,000 resamples, percentile intervals), the stricter outcome of regular KFRE use only (fitted by Firth because none of the 40 respondents with less-than-good guideline familiarity reported regular use, producing complete separation), KFRE selection in the tool checklist (Q3) as an independent measure of the same behavior, and a model restricted to structural covariates omitting guideline familiarity, which is associated with practice setting. The same six covariates were then applied to three secondary outcomes, which measure the use of other tools and are therefore reported as separate analyses rather than as sensitivity analyses of KFRE use: use of any nephrology-specific tool, use of the International IgA Nephropathy Prediction Tool, and use of the Mayo Imaging Classification for ADPKD; the last two were fitted by Firth because of the small number of events. Results are given in Tables S4–S6 (KFRE use) and Table S7 (other tools).

Standardization. Because university/academic respondents were over-represented relative to the national workforce, key estimates were recomputed as if the national mix of practice settings applied. Because the national distribution of practice settings is not published (see above), the sample was stratified into academic and non-academic respondents, the proportion of interest was estimated within each stratum, and the two stratum-specific proportions were recombined as a weighted average using hypothetical national academic shares of 20%, 30% and 40% as weights — a range chosen to bracket the reconstruction above, so that the conclusion can be read off at every plausible weight rather than resting on one. The observed sample proportion (an academic weight of 61.8%) is reported alongside for comparison. Confidence intervals for the standardized proportions were obtained from 2,000 stratified bootstrap resamples, drawn within strata to preserve the observed stratum sizes (Tables S2–S3).

Exploratory subgroup analysis. Respondents with at least good guideline familiarity who also agreed that prediction models improve the quality of care (n = 73) were compared by KFRE use across barrier, attitude, competence and practice characteristics using Fisher's exact test, with correction for the 21 comparisons made (Table S16). This analysis was prompted by peer review and is exploratory.

A two-sided P-value < 0.05 was considered statistically significant. Analyses were performed in R 4.5.2 (DescTools 0.99.60 for binomial and multinomial confidence intervals and for ordinal association measures; logistf 1.26.1 for penalized likelihood) and jamovi 2.6.26.0. Figures were drafted using Microsoft Excel® (Microsoft 365) and the UpSetR 1.4.0 package, and prepared for publication in Python 3.13 using Matplotlib 3.10.

Supplementary Results

This section expands on findings for which space in the main text was limited; data already reported in the main text are not repeated here.

Respondent Characteristics

The 131 respondents spanned a wide range of experience, with a predominance of early-career nephrologists (≤5 years of practice or in training, 28.2%) over those with more than three decades of experience (9.2%). Beyond CKD stage G3–G5 non-dialysis and hemodialysis patients reported in the main text, more than half of respondents reported caring primarily for geriatric patients aged >75 years (55.7%), and substantial proportions managed acute kidney injury (54.2%), kidney transplant recipients (40.5%), and peritoneal dialysis patients (28.2%). Full characteristics, including practice setting and patient populations, are detailed in Table S1. Beyond the KFRE, prognostic approaches in nephrology include multi-trajectory CKD progression models[S1]; the KFRE itself has been validated and recalibrated across European cohorts[S4,S5] and applied to guide primary-care referral[S11], while disease-specific tools underpin risk stratification in IgAN[S2] and ADPKD[S3].

Clinical Needs

In addition to the most frequently selected outcomes reported in the main text, respondents also valued predicting severe infections after kidney transplantation (45.8%), bleeding after kidney biopsy (42.7%), cardiovascular events in non-dialysis CKD (37.4%), peritoneal dialysis–associated peritonitis (36.6%), cardiovascular death in dialysis patients (36.6%), and all-cause mortality in dialysis patients (35.1%); the full ranking is given in Table 1 of the main text. This breadth of perceived needs, including outcomes relevant to patient-centered priorities, aligns with prior reports that patients and nephrologists value prediction across diverse outcomes.[S12] Among the setting-based differences, non-academic nephrologists more often valued predicting vascular-access complications (arteriovenous fistula dysfunction odds ratio 6.6 [95% CI: 2.8–15.5], $P < 0.001$; catheter-related infection 4.0 [1.7–9.4], $P = 0.001$), whereas academic nephrologists prioritized immunosuppression- and transplant-related outcomes, including severe infection after transplantation (2.9 [1.4–6.2], $P = 0.005$), transplant rejection (5.2 [2.3–11.5], $P < 0.001$), and severe infection after immunosuppressive treatment in glomerulonephritis (4.4 [2.0–9.4], $P < 0.001$). The needs–use discrepancy was most pronounced for kidney failure prediction, where dedicated tools could also inform vascular-access planning yet remain underused.[S12]

Implementation Barriers

Technological barriers are summarized in Table S9. Respondents most commonly regarded the absence of a single website giving easy access to multiple prediction tools as a key obstacle to implementing prediction models in their practice. This barrier was reported more often by non-academic than academic nephrologists (70.0% vs. 50.6%, $P = 0.03$); however, the difference did not survive correction for multiple comparisons, and technological barriers were otherwise distributed similarly across sex and practice setting.

Attitudes Toward Prediction Models

Although most respondents endorsed the trustworthiness and usefulness of prediction models (detailed in the main text and Table S10), the picture for local applicability was more equivocal. While 63% believed predictive models are sufficiently valid for the Polish population, nearly one-third (31.3% [95% CI: 24.0–39.7]) had no opinion on this issue. This perceived validity contrasts with the available evidence: the KFRE has been externally validated and recalibrated in several European cohorts,[S4,S5] but, to our knowledge, not in a Polish population—so endorsement of local validity may rest on extrapolation from other European populations rather than direct evidence. No statistically significant differences in any attitude item were observed across gender, professional experience, or practice setting (all adjusted $P > 0.05$).

Attitudes, Knowledge and Skill versus Barriers

Treating frequency of KFRE use as an ordinal outcome, the strongest associations were with knowledge and skill, not with attitudes or barriers (Table S14). Familiarity with the KDIGO 2024 guideline gave $\gamma = 0.70$ (95% CI: 0.54–0.85) and self-rated ability to interpret model output $\gamma = 0.62$ (95% CI: 0.45–0.79), whereas the four attitude items gave γ between 0.06 and 0.22 with every interval including zero. Familiarity and interpretive competence are correlated but distinct (Spearman $\rho = 0.59$): among the 80 respondents with at least good familiarity, only 23.8% rated themselves able to explain model output to a patient, and use tracked competence closely across its full scale (Table S15). The perceived barriers were only weakly associated with use, and in a direction the design cannot resolve: the composite severity of the organizational barriers gave $\gamma = -0.28$ (95% CI: −0.44 to −0.13) and the count of technological barriers $\gamma = -0.03$ (95% CI: −0.26 to 0.20). Whether the organizational association reflects barriers depressing use, or use softening the perception of barriers, cannot be established from cross-sectional data.

To hold knowledge and attitude constant, we defined an “informed and favorably disposed” subgroup — at least good guideline familiarity plus agreement that prediction models improve

the quality of care (n = 73) — and compared users of the KFRE with non-users (Table S16). No single perceived barrier distinguished the groups, and financial barriers were rare in both; what separated them was self-rated interpretive competence (96.0% vs. 66.7% rating it at least good, P = 0.002), the only one of 21 comparisons to survive correction for multiple testing. Its direction cannot be established, as competence may be acquired through use as readily as it enables it.

Characteristics of Non-Users of Clinical Prediction Models

Non-users (11.5% of respondents) were identified through two questionnaire items (8 through Q3 and 14 through Q4; Supplementary Methods). Beyond working more often outside academic hospitals (reported in the main text), non-users more frequently rated their familiarity with the KDIGO 2024 guideline as poor (20.0% vs. 0.9%, P = 0.04; Table S12). On attitudes, non-users appeared more likely to disagree that models increase decision confidence, but although this reached nominal significance (raw P = 0.022), it did not survive correction for multiple comparisons (adjusted P = 0.088); no other attitudinal differences were identified (Table S13). Non-users also more commonly rated their ability to interpret model results as very poor or declined to answer (Table S11; P = 0.003). Notably, every non-user expressed interest in training, indicating the gap is addressable.

Training Needs and Preferences

Beyond the most requested training topics and the overall preference for self-paced online courses reported in the main text, preferred training formats were self-paced (asynchronous) online courses (64.1% [95% CI: 55.6–71.8%]), on-the-job/workplace training (47.3% [95% CI: 39.0–55.8%]), practical workshops during conferences (45.0% [95% CI: 36.8–53.6%]), and self-study materials (44.3% [95% CI: 36.1–52.8%]). Because most respondents endorsed several formats (Figure S2), no single modality would reach all nephrologists. Among the 128 respondents interested in training (three reported needing none), a cumulative coverage analysis showed that self-paced online courses, on-the-job training, and practical workshops at conferences together would reach 92.2%, and adding self-study materials would raise coverage to 96.9%; live webinars and expert mentoring or consultations contributed least—the former because it largely overlapped with other formats, the latter because it was rarely selected.

Workforce Age Structure and Reconstructed Respondent Age

Age is the only demographic characteristic of Polish nephrologists reported nationally. The Supreme Audit Office (2024) gives the age structure of the 1,457 specialty holders as 0.5% under 35 years, 12.6% aged 36–45, 37.3% aged 46–55, 29.5% aged 56–65, and 20.2% over

65,[S8] so 13% are aged 45 or younger. We did not record age: in a specialty practiced by an estimated 800–950 physicians, a combination of age, sex and practice setting could identify an individual, so the questionnaire collected only the characteristics needed for the prespecified comparisons, using years of nephrology experience in place of age. To assess representativeness by age, we reconstructed the plausible age of each experience band from the structure of Polish specialty training, which brackets entry to nephrology between about 26 years of age under the current modular program and about 32 under the former two-step pathway (via internal medicine); the implied age of each experience band is given in Table S17.

Two limits, both pulling the same way, constrain the comparison. The published structure describes all specialty holders rather than the 800–950 who practice, and since attrition is probably concentrated at the older end the national share aged 45 or younger is higher than 13%—at most 23.8% if the entire difference is removed from the oldest bands. On our side, the latest-entry column is not a hard upper bound on age: a respondent who counted experience from certification rather than from entry to training, or whose career was interrupted, is older than it implies. The two profiles therefore lie closer together than a naive comparison suggests, by an amount the available data do not allow us to quantify. What is not in doubt is that our sample skews young: 28.2% reported fewer than five years of experience or being in training, and physicians in training were eligible while being absent from the register altogether. The sex distribution of Polish physicians within a given specialty is not published, so our 63.4% female proportion cannot be benchmarked.

Supplementary Discussion

Clinical Recommendations on Prediction Models in Poland

The strong association between guideline familiarity and use points to education, but the more basic constraint in Poland is the absence of any specialty-level recommendation that introduces prediction models into nephrology practice. The only national document that names the KFRE is a set of primary-care recommendations issued in 2024 by the National Consultants in Nephrology and in Family Medicine and the President of the Polish Society of Nephrology, which relayed the KDIGO recommendation to use validated calculators such as the KFRE in CKD G3–G5 and linked to an online one—before this survey opened.[S6] That document is, however, addressed to family physicians, and it stops at naming the tool: its own indications for nephrology consultation are framed entirely in terms of eGFR, albuminuria and clinical criteria, with no risk threshold among them, and it says nothing about how a physician should act on an estimated risk. At the time of the survey the KDIGO website carried no Polish translation of the 2024 guideline (only Italian), and no specialty-level Polish recommendation on prediction models addressed to nephrologists exists. Native-language guidance thus reached primary care before it reached the specialty from whose guideline it derives, without specifying how the resulting estimate should inform clinical decisions.

Actionability of a Risk Estimate in the Polish Setting

In Poland, nephrology is not among the specialties accessible without a referral, and referrals to nephrology outpatient clinics are issued by physicians of the public health-insurance system, usually the family physician. In this setting, the thresholds published alongside the KFRE are addressed to those physicians and can improve the appropriateness of referrals to specialist nephrology care.[S11] Our respondents receive these referrals rather than issue them. However much their use of the KFRE increased, the referral rate could not change as a direct consequence. The decision a Polish nephrologist does control is the reverse one—whether a stable, low-risk patient continues in specialist follow-up. This use of a risk estimate, risk-based de-escalation, has been implemented and evaluated abroad.[S13] No Polish cohort has been used to check either a threshold or the safety of acting on one.

Guidance for this reverse decision is, moreover, underdeveloped everywhere. A structured review of seven major CKD guidelines found that, whereas referral criteria are well established, standardized discharge criteria are rarely defined; where they exist they rest mostly on eGFR, albuminuria and sustained stability rather than a predicted risk, and only the UK Kidney Association attaches an explicit KFRE threshold—a low predicted failure risk (KFRE <3%)—

to a return to primary care.[S14] The KDIGO 2024 guideline is itself asymmetric in this respect: it offers KFRE thresholds for escalating care—a 5-year risk of 3–5% for nephrology referral, a 2-year risk >10% for adding multidisciplinary care, and a 2-year risk >40% for kidney-replacement-therapy modality education, preparation and vascular-access planning, and transplant referral (Practice Points 2.2.1–2.2.3)—but defines no risk threshold for the reverse step of discharge, and does not tie the KFRE to the frequency of monitoring, which it continues to structure by GFR and albuminuria category.[3]

Once a patient is under specialist care, a KFRE estimate can inform decisions the nephrologist does control: the onward referral for multidisciplinary advanced CKD consultations,[S15] the planning of kidney replacement therapy in advanced CKD (where a 2-year KFRE performs well even in older patients, in whom competing mortality risk is high),[S16] the timing of arteriovenous access creation,[8,S12] and the prioritization and timing of transplant evaluation and pre-emptive waitlisting.[S17] Across all of these uses, however, the evidence that acting on a KFRE estimate changes patient outcomes remains limited and largely single-centre,[S18] and none of the thresholds has been validated in a Polish population—precisely the gap a locally validated and recalibrated KFRE would fill.

Future Directions

Three lines of work follow from these findings. First, the KFRE requires validation and recalibration in a Polish cohort before any threshold-based use can be strongly recommended here. Second, because knowledge and interpretive skill separated users from non-users more than any barrier did, we intend to develop training in the formats respondents preferred—chiefly self-paced online courses, complemented by practical workshops and on-the-job training—and to evaluate its effect on the use and interpretation of prediction models, alongside a single Polish-language platform giving easy access to the recommended tools. Third, we plan to repeat this survey, built on an established implementation framework rather than as a descriptive item set, so that determinants the present instrument omitted can be measured—in particular whether clinicians use prediction tools when deciding which patients to retain in or discharge from specialist care, whether the tools support those decisions, whether they improve patients' acceptance of them, and whether clinicians prefer their own clinical judgment to a model-based estimate.

Supplementary Tables

Table S1. Study population characteristics (n = 131).

Characteristic	n (%)
**Gender**	
Women	83 (63.4)
Men	48 (36.6)
**Years of nephrology experience**	
<5 years or currently in training	37 (28.2)
5–10 years	27 (20.6)
11–20 years	30 (22.9)
21–30 years	25 (19.1)
>30 years	12 (9.2)
**Primary practice setting**	
University/academic hospital	81 (61.8)
Dialysis center or PD outpatient clinic	24 (18.3)
Non-university hospital	16 (12.2)
Other	10 (7.6)
**Primary patient population**[a]	
CKD stage G3–G5 non-dialysis	111 (84.7)
Hemodialysis	99 (75.6)
Geriatric patients (>75 years)	73 (55.7)
Acute kidney injury	71 (54.2)
Kidney transplant	53 (40.5)
Peritoneal dialysis	37 (28.2)

[a] Multiple responses allowed. Abbreviations: CKD, chronic kidney disease; PD, peritoneal dialysis.

Table S2. Key estimates stratified by practice setting.

Estimate	Academic	Non-academic	P
KFRE: any use	43/79 (54.4%)	22/45 (48.9%)	0.58
KFRE: regular use	10/79 (12.7%)	5/45 (11.1%)	>0.99
KFRE: selected in the tool checklist	31/79 (39.2%)	17/44 (38.6%)	>0.99
International IgAN Prediction Tool	32/79 (40.5%)	8/44 (18.2%)	0.015
Mayo Imaging Classification (ADPKD)	26/79 (32.9%)	6/44 (13.6%)	0.020
Use of any nephrology-specific tool	54/79 (68.4%)	23/44 (52.3%)	0.08
At least good familiarity with KDIGO 2024	57/79 (72.2%)	27/45 (60.0%)	0.17

P values from Fisher's exact test, unadjusted for multiple comparisons.

Table S3. Key estimates directly standardized to hypothetical national distributions of practice setting.

Estimate	Estimated % of nephrologists working in an academic setting in Poland			Observed (61.8% academic)
	20%	30%	40%	
Regular KFRE use	11.4 (4.3–19.3)	11.6 (5.4–18.6)	11.7 (5.9–18.6)	12.1
Any KFRE use	50.0 (38.6–61.7)	50.6 (40.0–61.0)	51.1 (41.6–60.6)	52.4
International IgAN Prediction Tool	22.6 (13.6–32.8)	24.9 (16.6–33.5)	27.1 (19.5–35.4)	32.5
Mayo Imaging Classification (ADPKD)	17.5 (10.0–26.6)	19.4 (11.9–27.7)	21.3 (14.4–29.0)	26.0
Use of any nephrology-specific tool	55.5 (43.3–67.4)	57.1 (46.3–67.0)	58.7 (49.0–68.3)	62.6
At least good familiarity with KDIGO 2024	62.4 (50.2–73.9)	63.6 (53.6–74.1)	64.9 (55.7–73.9)	67.7

Values are percentages with 95% confidence intervals from 2,000 stratified bootstrap resamples. The national distribution of practice settings among Polish nephrologists is not published; estimates are therefore reported across a plausible range of academic shares (the reconstruction in the Supplementary Methods).

Table S4. Predictors of Kidney Failure Risk Equation (KFRE) use: primary multivariable logistic regression model (n = 123; 65 events).

Covariate (contrast)	KFRE use, n/N (%)	Univariable analysis		Multivariable analysis	
		Crude OR (95% CI)	P	Adjusted OR (95% CI)	P
At least good familiarity with the KDIGO 2024 CKD guideline (vs. moderate/poor)	57/84 (67.9) vs. 8/40 (20.0)	8.44 (3.43–20.77)	<0.001	8.66 (3.54–23.44)	<0.001
Fully integrated electronic health record (vs. limited, partial or none)	37/60 (61.7) vs. 28/63 (44.4)	2.01 (0.98–4.13)	0.057	1.73 (0.75–4.04)	0.20
Women (vs. men)	43/79 (54.4) vs. 22/45 (48.9)	1.25 (0.60–2.60)	0.55	1.35 (0.58–3.19)	0.49
>10 years of nephrology experience (vs. ≤10)	35/62 (56.5) vs. 30/62 (48.4)	1.38 (0.68–2.80)	0.37	1.09 (0.46–2.60)	0.84
>40 CKD patients seen per week (vs. ≤40)	37/68 (54.4) vs. 28/56 (50.0)	1.19 (0.59–2.42)	0.63	0.94 (0.39–2.21)	0.89
University/academic hospital (vs. other setting)	43/79 (54.4) vs. 22/45 (48.9)	1.25 (0.60–2.60)	0.55	0.97 (0.39–2.38)	0.95

Outcome: any reported KFRE use (Q2 "I use it regularly" or "I know it but use it rarely") versus no use, among respondents with internally consistent answers to Q2–Q4 and complete data on all covariates. All covariates were prespecified and entered simultaneously; no stepwise selection was used. Model likelihood-ratio $P < 0.001$; McFadden pseudo-R^2 = 0.174. Crude estimates use all respondents with non-missing data for the covariate concerned (n = 123–124).

Table S5. Predictors of KFRE use: sensitivity model omitting guideline familiarity (structural covariates only; n = 123).

Covariate (contrast)	Adjusted OR (95% CI)	P
University/academic hospital (vs. other setting)	1.34 (0.60–2.98)	0.48
Women (vs. men)	1.25 (0.58–2.70)	0.57
>10 years of nephrology experience (vs. ≤10)	1.36 (0.63–2.95)	0.44
Fully integrated electronic health record	1.81 (0.86–3.87)	0.12
>40 CKD patients seen per week (vs. ≤40)	1.21 (0.56–2.61)	0.63

Guideline familiarity is plausibly a mediator of the association between practice setting and KFRE use and is measured by self-report on an item adjacent to the outcome; this model omits it. Conclusions are unchanged: practice setting remains unassociated with KFRE use.

Table S6. Sensitivity analyses for the association between guideline familiarity and KFRE use.

Analysis	n (events)	Adjusted OR (95% CI)	P
Primary model, Firth penalized likelihood	123 (65)	7.46 (3.17–19.22)	<0.001
Primary model, non-parametric bootstrap (percentile, 1,000 resamples)	123 (65)	10.02 (3.97–37.00)	—
Outcome: KFRE selected in the Q3 tool checklist	122 (48)	7.59 (2.80–24.76)	<0.001
Outcome: regular KFRE use only (Firth; complete separation)	123 (15)	16.55 (2.07–2,143.9)	0.003

Each row re-estimates the association between at least good guideline familiarity and KFRE use from the primary six-covariate model (Table S4), under a different estimator or a different operationalization of the same outcome; the adjusted odds ratio shown is for guideline familiarity. Firth denotes penalized likelihood, reported with penalized profile-likelihood intervals.

Table S7. Predictors of the use of other prediction tools.

Analysis	n (events)	Estimate of interest	Adjusted OR (95% CI)	P
Use of any nephrology-specific tool	122 (77)	Guideline familiarity	4.25 (1.84–10.17)	0.001
	122 (77)	Academic setting	1.88 (0.78–4.62)	0.16
International IgAN Prediction Tool (Firth)	122 (40)	Academic setting	3.24 (1.30–8.75)	0.011
Mayo Imaging Classification (Firth)	122 (32)	Academic setting	2.91 (1.04–9.13)	0.042
	122 (32)	Guideline familiarity	4.65 (1.51–18.75)	0.006
	122 (32)	Women	0.30 (0.12–0.72)	0.008

Tables S4–S7 contain the same six prespecified covariates. In the model for regular KFRE use, none of the 40 respondents with less-than-good guideline familiarity reported regular use, producing complete separation; that model and the two disease-specific models were fitted by Firth penalized likelihood and are reported with penalized profile-likelihood intervals. Abbreviations: IgAN, immunoglobulin A nephropathy.

Table S8. Organizational barriers to implementing predictive models.

Item	Answer				
	**Very significant barrier**	**Significant barrier**	**Moderate barrier**	**Minor barrier**	**Not a barrier**
Lack of time during patient visits, n (%)	62 (47.3)	39 (29.8)	24 (18.3)	4 (3.1)	2 (1.5)
Lack of institutional support (e.g., resources or procedures on when and how to use predictive models), n (%)	28 (21.4)	55 (42.0)	26 (19.8)	11 (8.4)	11 (8.4)
Lack of additional payment for risk prediction, n (%)	5 (3.8)	14 (10.7)	28 (21.4)	22 (16.8)	62 (47.3)

Survey item: "To what extent do the following organizational factors constitute a barrier to implementing predictive models?"

Table S9. Technological barriers to implementing predictive models.

Technological barrier	n (%)
Lack of automatic integration with the electronic health record system	97 (74.0)
Absence of a website enabling easy use of multiple prediction tools	76 (58.0)
Lack of internet access (online risk calculators) during patient visits	19 (14.5)
Insufficient technical skills or lack of technical support	11 (8.4)
No technological barriers perceived	12 (9.2)
Other	4 (3.1)

Survey item: "What are the main technological barriers to implementing predictive models in your practice?" (multiple selection allowed; n = 131).

Table S10. Attitudes toward prediction models.

Item	Answer				
	**Strongly agree**	**Somewhat agree**	**No opinion**	**Somewhat disagree**	**Strongly disagree**
"I trust the results of selected predictive models in making clinical decision", n (%)	24 (18.3)	86 (65.6)	16 (12.2)	4 (3.1)	1 (0.8)
"Predictive models are sufficiently valid for the Polish population", n (%)	10 (7.6)	73 (55.7)	41 (31.3)	7 (5.3)	0
"Using predictive models can increase my confidence in clinical decisions", n (%)	37 (28.2)	81 (61.8)	9 (6.9)	4 (3.1)	0
"Using predictive models can improve the quality of patient care", n (%)	43 (32.8)	72 (55.0)	12 (9.2)	4 (3.1)	0

Survey item: "To what extent do you agree with the following statements?"

Table S11. Self-rated knowledge in interpreting prediction model results, by user status.

Answer	All<br>n (%; 95% CI)	Non-users<br>(n = 15)	Users<br>(n = 115)	Standardized residuals *
Very good - I can interpret and explain to patients	20 (15.3; 6.9–24.2)	1 (6.7)	19 (16.5)	−0.995
Good - I understand basic results	64 (48.9; 40.5–57.8)	4 (26.7)	60 (52.2)	−1.855
Moderate - I need support in interpretation	31 (23.7; 15.3–32.6)	5 (33.3)	25 (21.7)	1.002
Poor - I have difficulties with interpretation	7 (5.3; 0–14.3)	0	7 (6.1)	−0.982
Very poor - I cannot interpret results	3 (2.3; 0–11.2)	2 (13.3)	1 (0.9)	3.024
I prefer not to answer this question	6 (4.6; 0–13.5)	3 (20.0)	3 (2.6)	3.019

Survey item: "How would you rate your knowledge in interpreting the results of predictive models?" One respondent was not included in the groups comparison due to inconsistent answers. * the common cutoffs for standardized residuals are 2 and −2 (values beyond these thresholds signal significant association between variables).

Table S12. Characteristics comparison of non-users and users of clinical prediction models.

Characteristics	Non-users<br>(n = 15)	Users<br>(n = 115)	P<br>(raw)	P<br>(adjusted)
**Woman gender**	11 (73.3%)	72 (62.6%)	0.416	0.832
**Familiarity with the KDIGO 2024 Guideline for CKD**			0.012	0.042
Very good	2 (13.3%)	17 (14.8%)		
Good	6 (40.0%)	64 (55.7%)		
Medium	4 (26.7%)	33 (28.7%)		
Poor	3 (20.0%)	1 (0.9%)		
**Working in university/academic hospital**	5 (33.3%)	76 (66.1%)	0.014	0.042
**Number of patients seen per week**			0.854	0.854
≤ 20	2 (13.3%)	14 (12.2%)		
21-40	4 (26.7%)	37 (32.2%)		
41-60	4 (26.7%)	29 (25.2%)		
61-80	4 (26.7%)	17 (14.8%)		
81-100	0	9 (7.8%)		
≥ 101	1 (6.7%)	9 (7.8%)		
**Years of nephrology experience**			0.809	0.854
<5 years or currently in training	3 (20.0%)	34 (29.6%)		
5-10 years	3 (20.0%)	24 (20.9%)		
11-20 years	3 (20.0%)	26 (22.6%)		
21-30 years	4 (26.7%)	21 (18.3%)		
> 30 years	2 (13.3%)	10 (8.7%)		
**Access to an electronic medical records system in practice**			0.661	0.854
Yes, fully integrated system	6 (42.9%)	58 (50.4%)		
Yes, but with limited functionality	7 (50%)	51 (44.3%)		
Partially, only some functions	1 (7.1%)	5 (4.3%)		
No, mainly paper documentation	0	1 (0.9%)		
Not reported	1	0		

Table S13. Comparison of attitudes toward prediction models between their non-users and users.

Attitude	Non-users (n = 15)	Users (n = 115)	P (raw)	P (adjusted)
**"I trust the results of selected predictive models in making clinical decisions"**			0.066	0.13
Strongly agree/agree	11 (73.3)	99 (86.1)		
No opinion	2 (13.3)	14 (12.2)		
Strongly disagree/disagree	2 (13.3)	2 (1.7)		
**"Predictive models are sufficiently valid for the Polish population"**			0.263	0.26
Strongly agree/agree	8 (53.3)	74 (64.3)		
No opinion	5 (33.3)	36 (31.3)		
Strongly disagree/disagree	2 (13.3)	5 (4.3)		
**"Using predictive models can increase my confidence in clinical decisions"**			0.022	0.09
Strongly agree/agree	11 (73.3)	107 (93.0)		
No opinion	2 (13.3)	6 (5.2)		
Strongly disagree/disagree	2 (13.3)	2 (1.7)		
**"Using predictive models can improve the quality of patient care"**			0.095	0.13
Strongly agree/agree	11 (73.3)	104 (90.4)		
No opinion	3 (20.0)	9 (7.8)		
Strongly disagree/disagree	1 (6.7)	2 (1.7)		

Table S14. Association of guideline familiarity, interpretive competence, attitudes and barriers with the frequency of KFRE use.

Ordinal predictor	Goodman–Kruskal γ (95% CI)
Familiarity with the KDIGO 2024 CKD guideline	0.70 (0.54 to 0.85)
Self-rated ability to interpret model output	0.62 (0.45 to 0.79)
"I trust the results of selected predictive models in making clinical decisions"	0.22 (−0.02 to 0.47)
"Predictive models are sufficiently valid for the Polish population"	0.18 (−0.04 to 0.41)
"Using predictive models can increase my confidence in clinical decisions"	0.08 (−0.18 to 0.33)
"Using predictive models can improve the quality of patient care"	0.06 (−0.17 to 0.29)
Count of technological barriers cited (0–4; higher = more barriers)	−0.03 (−0.26 to 0.20)
Severity of organizational barriers (time, institutional support, payment; higher = more)	−0.28 (−0.44 to −0.13)

Outcome: frequency of KFRE use (Q2), treated as an ordinal variable with four levels (does not know the tool; has heard of it but does not use it; knows it but uses it rarely; uses it regularly); n = 124.

Table S15. KFRE use across the self-rated interpretive-competence scale.

Self-rated interpretive competence	n	Any KFRE use	Regular KFRE use
Can interpret results and explain to patients	19	78.9%	31.6%
Understands basic results	63	68.3%	12.7%
Needs support with interpretation	26	23.1%	3.8%
Finds interpretation difficult or impossible	10	0%	0%

Analysis restricted to the 118 respondents internally consistent on use who also answered the competence item.

Table S16. Users versus non-users of the KFRE among respondents who were both informed and favorably disposed (n = 73).

Characteristic	KFRE users (n = 51)	KFRE non-users (n = 22)	P
Perceived barriers			
Lack of time during visits: at least significant barrier	35 (68.6%)	17 (77.3%)	0.58
Lack of institutional support: at least significant barrier	28 (54.9%)	12 (54.5%)	>0.99
Lack of additional payment: at least significant barrier	6 (11.8%)	2 (9.1%)	>0.99
Reported absent integration with the electronic health record as a barrier	36 (70.6%)	17 (77.3%)	0.78
Reported absence of a single web platform for multiple tools as a barrier	29 (56.9%)	14 (63.6%)	0.62
Reported no internet access during visits as a barrier	9 (17.6%)	3 (13.6%)	>0.99
Reported insufficient technical skills or lack of technical support as a barrier	2 (3.9%)	2 (9.1%)	0.58
Perceives no technological barriers at all	6 (11.8%)	3 (13.6%)	>0.99
Practice and demographic characteristics			
Fully integrated electronic health record available in practice	31 (60.8%)	9 (40.9%)	0.13
University/academic hospital	33 (64.7%)	16 (72.7%)	0.59
>40 CKD patients seen per week	32 (62.7%)	13 (59.1%)	0.80
>10 years of nephrology experience	30 (58.8%)	11 (50.0%)	0.61
Women	34 (66.7%)	11 (50.0%)	0.20
Attitudes and clinical relevance			
Strongly agrees that models improve quality of care	20 (39.2%)	7 (31.8%)	0.61
Trusts the results of selected predictive models	47 (92.2%)	18 (81.8%)	0.23
Agrees models are sufficiently valid for the Polish population	33 (64.7%)	14 (63.6%)	>0.99
No opinion on validity for the Polish population	16 (31.4%)	7 (31.8%)	>0.99
Reported progression to kidney failure as an outcome useful to predict	40 (78.4%)	15 (68.2%)	0.39
Knowledge and skill			
Very good (rather than good) familiarity with KDIGO 2024	15 (29.4%)	2 (9.1%)	0.07
Can interpret model results and explain them to patients[a]	14 (28.0%)	4 (19.0%)	0.56
Self-rated interpretive competence at least good[a]	48 (96.0%)	14 (66.7%)	0.002

Subgroup: respondents with at least good familiarity with the KDIGO 2024 guideline who also agreed that prediction models improve the quality of patient care. "Users" reported using the KFRE regularly or rarely; "non-users" had heard of it but did not use it, or did not know it. P values from Fisher's exact test. Of the 21 comparisons made, only self-rated interpretive competence survives Bonferroni or Benjamini–Hochberg correction (adjusted P

= 0.04). The analysis is exploratory, was prompted by peer review, and is cross-sectional: clinicians who use the KFRE may rate their interpretive competence highly because they use it. The mean number of technological barriers cited was 1.49 (users) versus 1.64 (non-users), P = 0.56. [a] Two respondents declined the item on interpretive competence, giving denominators of 50 and 21 for the last two rows.

Table S17. Reconstruction of respondent age from years of nephrology experience.

Reported nephrology experience	n (%)	Implied age	
		**Earliest entry (26+ years)**	**Latest entry (32+ years)**
<5 years or in training	37 (28.2)	26–31	32–37
5–10 years	27 (20.6)	31–36	37–42
11–20 years	30 (22.9)	37–46	43–52
21–30 years	25 (19.1)	47–56	53–62
>30 years	12 (9.2)	≥57	≥63

Entry to nephrology is bracketed between about 26 years of age under the current modular program and about 32 under the former two-step pathway (via internal medicine). This is an approximation with the assumptions stated in the Supplementary Results, not a measured variable.

Supplementary Figures

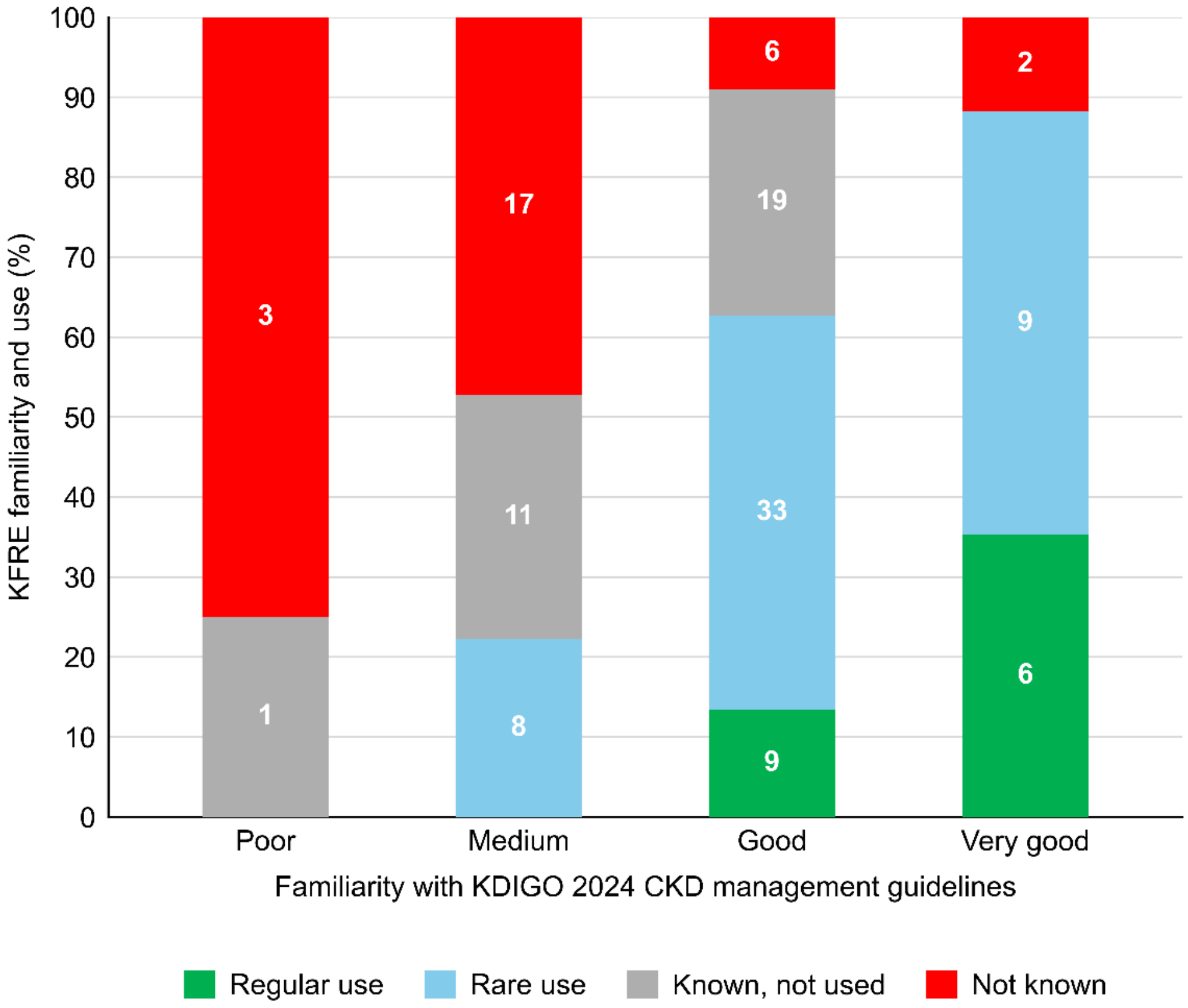

Figure S1. Correlation between self-reported familiarity with the KDIGO 2024 Clinical Practice Guideline for the Evaluation and Management of Chronic Kidney Disease and frequency of Kidney Failure Risk Equation (KFRE) use (n = 124).

Bars show the percentage distribution of KFRE familiarity and use within each familiarity category; numbers within segments are absolute counts.

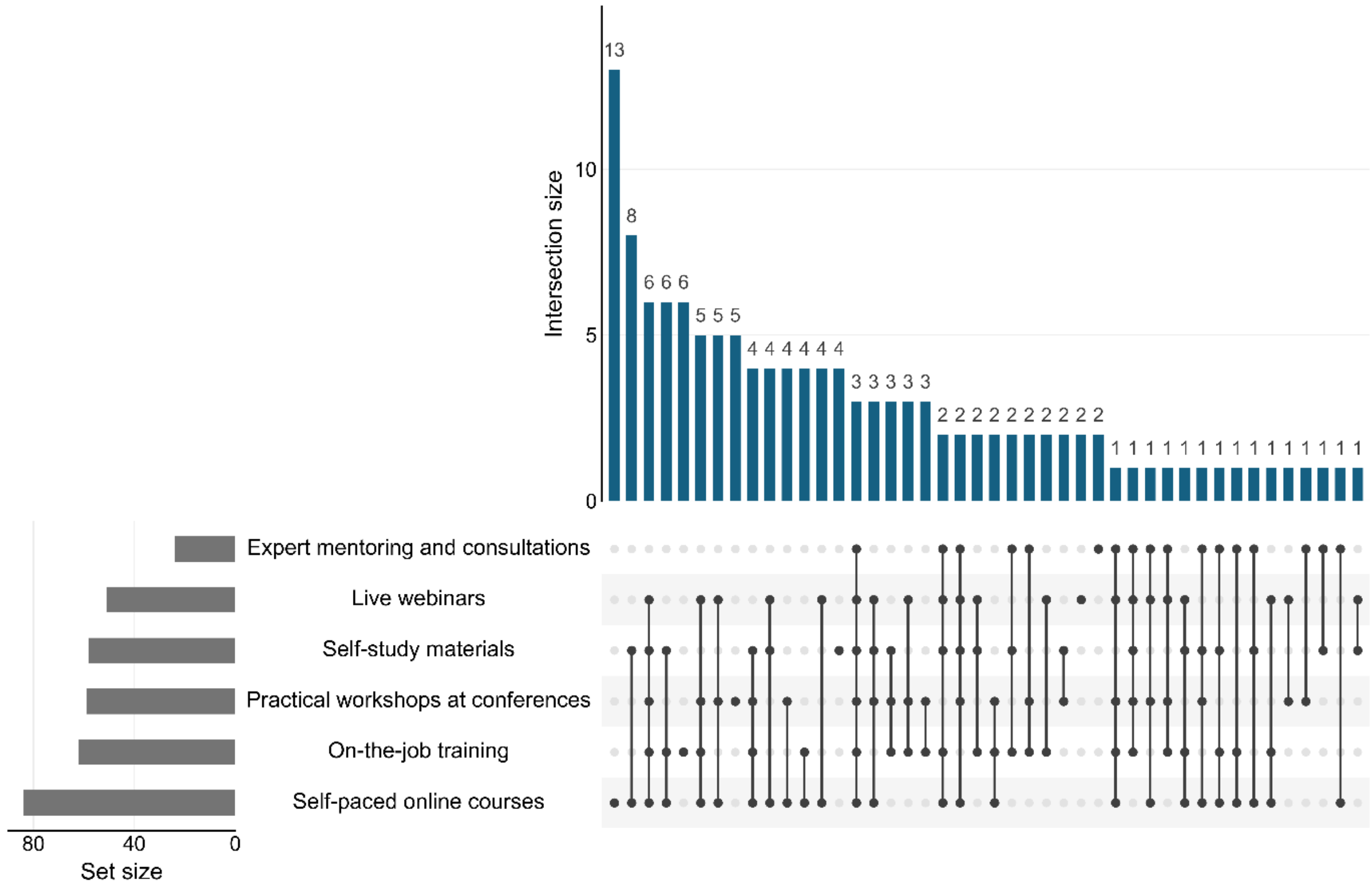

Figure S2. UpSet plot of preferred training methods (n = 128).

Horizontal bars (left) show the number of respondents selecting each format; the matrix indicates which formats constitute each combination; vertical bars (top) show the number of respondents selecting exactly that combination. Three respondents who reported needing no training are excluded.

Supplementary References

S1. Burckhardt P, Nagin DS, Padman R. Multi-Trajectory Models of Chronic Kidney Disease Progression. AMIA Annu Symp Proc. 2017;2016:1737.

S2. Rovin BH, Barratt J, Cook HT, et al. KDIGO 2025 Clinical Practice Guideline for the Management of Immunoglobulin A Nephropathy (IgAN) and Immunoglobulin A Vasculitis (IgAV). Kidney Int. 2025;108(4S):S1-S71. doi:10.1016/J.KINT.2025.04.004

S3. Devuyst O, Ahn C, Barten TRM, et al. KDIGO 2025 Clinical Practice Guideline for the Evaluation, Management, and Treatment of Autosomal Dominant Polycystic Kidney Disease (ADPKD). Kidney Int. 2025;107(2S):S1-S239. doi:10.1016/J.KINT.2024.07.009

S4. Bundó-Luque D, Cunillera-Puértolas O, Cobo-Guerrero S, et al. Recalibrating the kidney failure risk equation for a Mediterranean European population: reducing age and sex inequality. Front Med (Lausanne). 2024;11:1497780. doi:10.3389/FMED.2024.1497780

S5. Escamilla-Cabrera B, Montomoli M, Kislikova M, et al. Multicenter validation of the Kidney Failure Risk Equation (KFRE) formula in Spanish patients with advanced chronic kidney disease. Nefrologia (Engl Ed). 2025;45(5):388-396. doi:10.1016/J.NEFROE.2025.05.003

S6. Gellert R, Mastalerz-Migas A, Krajewska M, Ledwoch J. Wytyczne konsultanta krajowego w dziedzinie nefrologii, konsultanta krajowego w dziedzinie medycyny rodzinnej oraz prezesa Polskiego Towarzystwa Nefrologicznego dotyczące diagnostyki i leczenia przewlekłej choroby nerek w POZ, z uwzględnieniem opieki koordynowanej, z 19 lipca 2024 r. Lekarz POZ. 2024;10(6):383-399.

S7. Naczelna Izba Lekarska (Supreme Medical Chamber). Zestawienie liczbowe lekarzy i lekarzy dentystów wg dziedziny i stopnia specjalizacji, z uwzględnieniem podziału na lekarzy wykonujących i nie wykonujących zawodu [Numerical breakdown of physicians and dentists by field and level of specialization, by whether or not they practise the profession]. Data as of 31 December 2024 from the Central Register of Physicians and Dentists of the Republic of Poland (Centralny Rejestr Lekarzy RP). https://nil.org.pl/uploaded_files/1737380333_zestawienie-nr-4-31122024.pdf. Accessed August 17, 2026.

S8. Najwyższa Izba Kontroli (Supreme Audit Office). Diagnostyka i zapewnienie opieki nad pacjentami z przewlekłą chorobą nerek [Diagnosis and provision of care for patients with chronic kidney disease]. Report no. 131/2024/P/24/068/LKR. https://www.nik.gov.pl/plik/id,31243,vp,34339.pdf. Accessed August 17, 2026.

S9. Motyl M. Stan opieki nefrologicznej [The state of nephrology care]. Menedżer Zdrowia. March 19, 2025. https://www.termedia.pl/mz/Stan-opieki-nefrologicznej-,60878.html. Accessed August 17, 2026.

S10. Kraska W. Odpowiedź na interpelację nr 42118 w sprawie sytuacji w nefrologii [Reply to parliamentary interpellation no. 42118 on the situation in nephrology]. https://www.sejm.gov.pl/Sejm9.nsf/interpelacja.xsp?typ=INT&nr=42118. Accessed August 17, 2026.

S11. Li K, Pirabhahar S, Thomsett M, et al. Use of kidney failure risk equation as a tool to evaluate referrals from primary care to specialist nephrology care. Intern Med J. 2024;54(7):1126-1135. doi:10.1111/IMJ.16377

S12. Henriques A, Venda J, Ferreira E, Costa J, Oliveira N, Alves R. Risk prediction for hemodialysis initiation: The role of Kidney Failure Risk Equation and personalized timely vascular access referral. J Vasc Access. 2026;27(2):648-655. doi:10.1177/11297298251362913

S13. Che M, Iliescu E, Thanabalasingam S, Day AG, White CA. Death and Dialysis Following Discharge From Chronic Kidney Disease Clinic: A Retrospective Cohort Study. Can J Kidney Health Dis. 2022;9:20543581221118434. doi:10.1177/20543581221118434

S14. Oliva-Damaso N, Martinez-Palma L, Oliva-Damaso E, Payan J, Glassock RJ. Discharge criteria from nephrology follow-up back to primary care in modern-era CKD: a review of main guidelines. Clin Kidney J. 2026;19(4):sfag057. doi:10.1093/ckj/sfag057

S15. Gallego-Valcarce E, Rey-Cárdenas A, López-Melero E, et al. KFRE as a criterion for referral to advanced chronic kidney disease consultations. Nefrologia (Engl Ed). 2026;46(1):501412. doi:10.1016/j.nefroe.2026.501412

S16. Temprado Collado A, Toapanta N, Román Cabezas M, et al. Planning of kidney replacement therapy in advanced CKD using the KFRE formula in a Spanish multicenter cohort. Clin Kidney J. 2026;19(7):sfag192. doi:10.1093/ckj/sfag192

S17. Ku E, Copeland T, Chen LX, et al. Strategies to Guide Preemptive Waitlisting and Equity in Waittime Accrual by Race/Ethnicity. Clin J Am Soc Nephrol. 2024;19(3):292-300. doi:10.2215/CJN.0000000000000354

S18. Bhachu HK, Fenton A, Cockwell P, Aiyegbusi O, Kyte D, Calvert M. Use of the kidney failure risk equation to inform clinical care of patients with chronic kidney disease: a mixed-methods systematic review. BMJ Open. 2022;12(1):e055572. doi:10.1136/bmjopen-2021-055572
